# Comparative pharmacodynamic and pharmacokinetic characteristics of subcutaneous insulin glulisine and insulin aspart prior to a standard meal in obese subjects with type 2 diabetes

**DOI:** 10.1111/j.1463-1326.2010.01343.x

**Published:** 2011-03

**Authors:** G B Bolli, S Luzio, S Marzotti, F Porcellati, C Sert-Langeron, B Charbonnel, Y Zair, D R Owens

**Affiliations:** 1 Department of Internal Medicine, University of PerugiaPerugia, Italy; 2 Diabetes Research Unit, University Hospital LlandoughVale of Glamorgan, UK; 3 sanofi-aventisParis, France; 4 Centre de Recherche en Nutrition Humaine, INSERM U 915Nantes, France

**Keywords:** insulin analogues, insulin aspart, insulin glulisine, insulin therapy, obesity, obesity therapy, pharmacodynamics, pharmacokinetics, type 2 diabetes

## Abstract

**Aims:** A multinational, randomized, double-blind, two-way crossover trial to compare the pharmacokinetic and pharmacodynamic properties of bolus, subcutaneously administered insulin glulisine (glulisine) and insulin aspart (aspart) in insulin-naÏve, obese subjects with type 2 diabetes.

**Methods:** Thirty subjects [9/21 females/males; mean ± SD age: 60.7 ± 7.7 years; body mass index (BMI): 33.5 ± 3.3 kg/m^2^; duration of diabetes: 6.8 ± 4.6 years; HbA1c: 7.1 ± 0.8%] were included in the analysis. They fasted overnight and then received a 0.2 U/kg subcutaneous dose of glulisine or aspart 2 min before starting a standardized test meal, 7 days apart, according to a randomization schedule. Blood samples were taken every 15 min, starting 20 min before the meal and ending 6 h postprandially.

**Results:** The area under the absolute glucose concentration–time curve between 0 and 1 h after insulin injection and maximal glucose concentration was significantly lower with glulisine than with aspart (p = 0.0455 and 0.0337, respectively). However, for the total study period, plasma glucose concentration was similar for glulisine and aspart. Peak insulin concentration was significantly higher for glulisine than for insulin aspart (p < 0.0001). Hypoglycaemic events (≤ 70 mg/dl with or without symptoms) occurred in 13 and 16 subjects treated with glulisine and aspart, respectively, but there were no cases of severe hypoglycaemia requiring intervention.

**Conclusions:** Glulisine was associated with lower glucose levels during the first hour after a standard meal; the remaining glucose profiles were otherwise equivalent, with higher insulin levels observed throughout the study period.

## Introduction

The ultimate goal of therapy in type 2 diabetes (T2DM) is to achieve near-normoglycaemia [1]. The Global Task Force on Glycaemic Control recommended HbA1c levels of less than 6.5% as a good target for certain people with T2DM [2], although it also stated that HbA1c and blood glucose targets should be individualized, taking into account factors such as age, existing complications, risk of future complications, diabetes duration and risk of hypoglycaemia. Type 2 diabetes is generally characterized by the presence of relative insulin deficiency, including postprandial insulin deficiency [3], in the presence of insulin resistance. Therefore, an important facet of T2DM treatment is to support and/or supplement the insulin deficit to replicate as closely as possible the normal insulin secretory pattern, including an early response to a nutrient challenge. The time–action profile of subcutaneously injected regular human insulin (RHI) provides a slow onset of action, with a peak effect at 3 h after dosing and a relatively prolonged duration of action beyond 8 h [4]. This requires the insulin to be administered up to 1 h premeal in an attempt to accommodate these deficiencies.

In response to these limitations of RHI, three rapid-acting insulin analogues have been introduced: insulin aspart (aspart), insulin glulisine (glulisine) and insulin lispro (lispro). These analogues all have a rapid onset of action (within 30–60 min) and a peak action within 2 h to allow for appropriate control of postprandial glucose (PPG) fluctuations when given within 5 min preprandially [5]. Glulisine differs from RHI by the replacement of asparagine by lysine at position B3 and lysine by glutamic acid at B29 [6]. The modifications in glulisine allow it to exist as more stable dimers and monomers at pharmaceutical concentrations, allowing glulisine to be suspended in a zinc-free buffer, unlike RHI and other rapid-acting insulin analogues [6]. Lispro differs in that the lysine and proline residues at the C-terminal end of the B chain are reversed, which prevents the formation of insulin dimers and hexamers. Aspart differs in that the amino acid residue at position B28 is substituted with aspartic acid, which increases charge repulsion to inhibit the formation of hexamers [6].

Glulisine has been shown to have a more rapid onset of action and a shorter duration of action compared with RHI in obese subjects without diabetes [7]. In addition, glulisine was shown to have a faster onset of action in obese subjects without diabetes [8] and faster absorption with higher postprandial insulin levels in people with T2DM compared with lispro [9]. Similar findings have also been reported in healthy individuals [10] and individuals with type 1 diabetes (T1DM) [11,12]. A recent study in healthy individuals has also shown a more rapid onset of action for glulisine compared with aspart [13].

To date, however, no study has directly compared the pharmacokinetic (PK) and pharmacodynamic (PD) properties of glulisine with those of aspart in people with T2DM. Therefore, the aim of this study was to conduct such a study in obese subjects with T2DM with the comparative insulins given immediately before a standardized test meal.

## Materials and Methods

This was a multinational, randomized, double-blind, two-way crossover trial comparing the PK and PD characteristics of glulisine with those of aspart.

### Study Population

Obese [body mass index (BMI) 30–40 kg/m^2^] males or females aged 18–70 years with T2DM for at least 1 year, treated with oral hypoglycaemic agents (OHAs) for at least 6 months and with HbA1c levels of less than 8.5% were eligible for this study. Subjects were excluded if they had T1DM or were currently using insulin. Further exclusion criteria were pregnancy or breastfeeding, taking medications known to influence insulin sensitivity (e.g. corticosteroids), a history of acute metabolic complications in the past 3 months, recurrent severe hypoglycaemia or hypoglycaemia unawareness, impaired renal or hepatic function and any history of drug or alcohol abuse.

All subjects provided written informed consent and the study was approved by an independent ethics committee at each of the three study sites (Perugia, Italy; Nantes, France and Cardiff, UK).

### Study Design and Treatment

Subjects attended a screening visit, performed 1–2 weeks before the first study day, to confirm eligibility. At this visit, baseline characteristics, vital signs and laboratory tests (haematology, clinical chemistry, C-peptide level, HbA1c level and urinalysis) were evaluated after a 12-h fast. On the first study day, the subjects arrived at the respective research centres at approximately 8 a.m., after fasting and omitting their OHAs for 12 h before the visit. In accordance with the randomization scheme, subjects received a 0.2 U/kg dose of either glulisine or aspart subcutaneously within 2 min before starting a standardized meal (692 kcal: 54% carbohydrate, 17% protein and 28% lipid), which they had to finish within 30 min. After a 7-day washout period, the same procedure was repeated using the alternative insulin preparation.

Blood samples were collected at −20 and −10 min and immediately before the meal (0 min), every 10 min for the first 2 h after the meal and then every 15 min for the remaining 4-h period of the study. Plasma glucose, insulin, C-peptide (Invitron, Monmouth, UK) and non-esterified fatty acid (NEFA; Wako NEFA-C kit, Wako Chemicals, Neuss, Germany) levels were determined using validated techniques. Aspart (Capio Diagnostics AS, Copenhagen, Denmark) and glulisine (Linco Research, Missouri, USA) concentrations were determined using analogue-specific assay kits at a central laboratory. All adverse events and episodes of hypoglycaemia were recorded.

### Outcome Measures

The primary objective of this study was to assess the PD effect of glulisine compared with aspart on PPG excursions during the first hour after a standard meal, as measured by the area under the glucose concentration–time curve (AUC) between 0 and 1 h after insulin injection (AUC_0–1 h_). Secondary objectives included assessment of the PD effects of these insulins on PPG excursions up to 6 h after a standard meal (AUC_0–6 h_) and assessment of the postprandial insulin excursion after a standard meal in each treatment group. Other objectives were to evaluate C-peptide and NEFA levels in each treatment group.

### Statistical Analysis

Pharmacodynamic parameters were derived from the individual glucose concentration profiles and PK parameters from the serum aspart and glulisine concentrations. The AUCs were calculated according to the linear trapezoidal rule [14]. PK analyses were carried out using a non-compartmental approach in order to determine maximum insulin concentration (*C*_max_) and time to maximum insulin concentration (*T*_max_) parameters from serum insulin concentrations. Also, the incremental AUCs (0–1, 0–2, 0–4 and 0–6 h for PD and PK), maximum glucose concentration (GLU_max_), maximum incremental glucose excursion (ΔGLU_max_) and *C*_max_ were analysed by analysis of variance with subject, treatment, sequence group and period effects. Two-sided 90% confidence intervals (CIs) were calculated for the mean differences or mean ratios. Time to ΔGLU_max_ and time to fraction of total glucose AUC (10 and 20%) and corresponding PK parameters [*T*_max_ and time to fraction of total insulin AUC (10 and 20%)] were analysed using Wilcoxon's signed rank test and Hodges–Lehmann 90% CIs were calculated for the median difference, as previously described [15]. Superiority testing was carried out at the 5% significance level. For any given variable (except time measurements), glulisine and aspart were considered to be clinically similar if the difference between them was non-significant and if the two-sided 90% CIs for the ratios of the means were within 80–125%.

PK and PD analyses were performed in all subjects who completed the study with no major protocol deviations and who had data considered as evaluable. Safety (hypoglycaemia and adverse events) was assessed for all subjects who were exposed to study treatment.

## Results

### Subject Disposition

A total of 43 subjects were screened, of whom six were excluded because of having a BMI outside the predefined range (n = 2), an HbA1c level of more than 8.5% (n = 2), age over 70 years (n = 1) or taking prohibited medication (n = 1). Therefore, 37 subjects [mean (± standard deviation) age 60.3 ± 8.3 years, BMI 33.7 ± 3.3 kg/m^2^, diabetes duration 7.3 ± 4.9 years, HbA1c 7.1 ± 0.8%] were randomized. Of the 37 subjects randomized, seven were subsequently excluded from the PK and PD analyses: one for premature withdrawal after the first study day (having received aspart) and six for major protocol deviations [two subjects with medical conditions at inclusion who were erroneously included; one each for use of corticosteroids during the study, missing PK/PD values in the first hour after drug administration, unusable PK assessments (very low aspart plasma levels, incompatible with aspart administration) and duration of meal intake longer than 30 min (85 min)]. The latter two subjects were excluded after the database lock, following a recommendation by the Steering Committee. Therefore, 30 subjects were included in the final analysis and the baseline characteristics are represented in Table 1. There were no differences between the subjects included in the final analysis and all randomized subjects (data not shown). The mean doses of glulisine and aspart were 19.5 ± 2.7 and 19.4 ± 2.7 U, respectively.

**Table 1:** Baseline characteristics of the study subjects

	Sequence glulisine/aspart (n = 16)	Sequence aspart/glulisine (n = 14)	All (n = 30)
Females/males, n	3/13	6/8	9/21
Age, years*	61.2 ± 7.7	59.7 ± 8.3	60.7 ± 7.7
Weight, kg*	100.4 ± 16.1	94.1 ± 10.7	96.3 ± 14.3
Height, cm*	173.1 ± 8.6	166.3 ± 7.2	169.4 ± 8.7
BMI, kg/m^2^*	33.3 ± 3.4	34.0 ± 3.3	33.5 ± 3.3
Diabetes duration, years*	6.3 ± 4.0	7.5 ± 5.3	6.8 ± 4.6
HbA1c, %*	7.0 ± 0.8	7.2 ± 0.8	7.1 ± 0.8
Oral hypoglycaemic agents, n (%)	16 (100)	14 (100)	30 (100)
Biguanides	15 (3.8)	14 (100)	29 (96.7)
Sulphonylureas	5 (31.3)	9 (64.3)	14 (46.7)
Thiazolidinediones	3 (18.8)	3 (21.4)	6 (20.0)
Glinides	1 (6.3)	1 (7.1)	2 (6.7)

BMI, body mass index.

* Data are mean ± standard deviation.

### Pharmacodynamics

**Figure 1:**
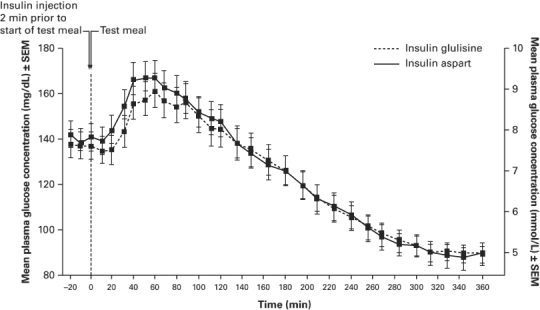
Mean plasma glucose concentrations over time. SEM, standard error of the mean.

Mean blood glucose levels at baseline were 137.4 ± 33.2 and 140.5 ± 32.5 mg/dl for the glulisine and aspart groups, respectively. The plasma glucose concentrations over time are shown in figure 1. Both mean AUC_0–1 h_ (149 vs. 158 mg·h/dl; p = 0.0455) and mean GLU_max_ (170 vs. 181 mg/dl; p = 0.0337) were significantly lower with glulisine than with aspart. Point estimates (glulisine/aspart) for AUC_0–1 h_ and GLU_max_ were 94% (90% CI: 90–99) and 94% (90% CI: 90–99), respectively (Table 2). No statistically significant differences were observed with baseline-subtracted data in any of the periods analysed (data not shown).

**Table 2:** Pharmacodynamic and pharmacokinetic results

	Estimated sample mean (n = 30)			Estimate and 90% CI for mean ratios* (glulisine/aspart)	Estimate and 90% CI for mean differences§ (glulisine/aspart)
			
	Glulisine	Aspart	p value		
*Pharmacodynamics results*					
AUC_0–1 h_ (mg·h/dl)	149	158	0.0455	94% (90–99)	—
AUC_0–6 h_ (mg·h/dl)	738	750	0.5382	98% (95–104)	—
AUC_0–1 h_/AUC_0–6 h_ (%)	20	21	0.0334	95% (92–99)	—
AUC_0–2 h_/AUC_0–6 h_ (%)	41	42	0.0341	96% (94–99)	—
AUC_0–4 h_/AUC_0–6 h_ (%)	74	75	0.0912	99% (97–100)	—
ΔGLU_max_ (mg/dl)	33	40	0.0634	81% (70–100)	−8 (−15 to −10)
GLU_max_ (mg/dl)	170	181	0.0337	94% (90–99)	−11 (−19 to −3)
Time to ΔGLU_max_ (min)	60.0†	59.5†	0.3328	—	−5 (−20 to 5)¶
Time to 10% of total glucose AUC (min)	40.0†	40.0†	0.3566	—	−2 (−6 to 2)¶
Time to 20% of total glucose AUC (min)	67.5†	65.0†	0.9681	—	0 (−4 to 3)¶
*Pharmacokinetics results*					
AUC_0–1 h_ (pmol·h/l)	272 (297)‡	138 (167)‡	<0.0001	197% (157–248)	—
AUC_0–6 h_ (pmol·h/l)	2002 (2077)‡	1289 (1333)‡	<0.0001	155% (141–171)	—
AUC_0–1 h_/AUC_0–6 h_ (%)	14 (2.6)‡	11 (2.4)‡	0.0340	127% (106–152)	—
AUC_0–2 h_/AUC_0–6 h_ (%)	36 (3.6)‡	35 (3.6)‡	0.5566	103% (95–110)	—
AUC_0–4 h_/AUC_0–6 h_ (%)	78 (4.3)‡	77 (4.3)‡	0.3716	101% (99–103)	—
*C*_max_ (pmol/l)	534 (570)‡	363 (385)‡	<0.0001	147% (133–163)	—
Time to fraction of total insulin AUC (10%) (min)	60.0†	60.5†	0.0372	—	−12 (−26 to −1)¶
Time to fraction of total insulin AUC (20%) (min)	90.0†	91.0†	0.9109	—	0 (−12 to 14)¶
*T*_max_ (min)	120.0†	93.0†	0.5133	—	17 (−10 to 37)¶

CI, confidence interval; AUC_0–X h_, area under the curve for the period 0–X h; ΔGLU_max_, maximum glucose excursion; GLU_max_, peak glucose concentration; *C*_max_, peak insulin concentration; *T*_max_, time to peak insulin concentration.

* For pharmacodynamic parameters, point estimate and 90% CI for the ratio of treatment means according to Fieller's Theorem, based on untransformed data. For pharmacokinetic parameters, point estimate and 90% CI for the ratios of the treatment means, based on ln-transformed data.

† Data are median.

‡ Data are sample geometric mean (arithmetic mean).

§ Point estimate and 90% CI for the difference of treatment means, from parametric data analysis (analysis of variance), based on untransformed data.

¶ Point estimate and 90% CI for the difference of treatment medians from non-parametric analysis (Hodges and Lehmann method).

The AUC ratios for AUC_0–1 h_/AUC_0–6 h_ (p = 0.0334) and AUC_0–2 h_/AUC_0–6 h_ (p = 0.0341) were significantly lower for glulisine than aspart, with point estimates of 95% (90% CI: 92–99) and 96% (90% CI: 94–99), respectively (Table 2). Moreover, taking into account the total study duration (6 h), the overall plasma glucose concentration was similar between groups treated with glulisine and aspart.

Mean C-peptide plasma concentration profiles were similar after glulisine and aspart injections (data not shown), with maximum concentrations of 2.08 and 2.07 pmol/ml, respectively, occurring at 90 min for both insulin analogues.

Mean NEFA concentrations decreased from 0.50 to 0.11 mmol/l at 180 min with glulisine and from 0.51 to 0.11 mmol/l at 120 min with aspart; the NEFA concentrations then increased to 0.32 and 0.31 mmol/l with glulisine and aspart, respectively.

### Pharmacokinetics

**Figure 2:**
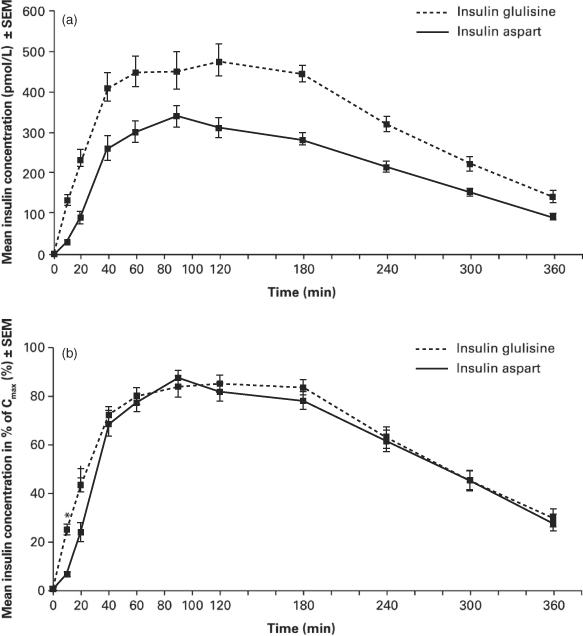
(a) Mean plasma insulin concentrations over time and (b) mean plasma insulin concentrations in percentage of peak insulin concentration over time. ^*^p < 0.001 compared with insulin aspart at 10 min and † p > 0.001 compared with insulin aspart at 20 min. SEM, standard of the mean; *C*_max_, peak insulin concentration.

Table 2 also represents the PK results derived from the insulin concentration profiles illustrated in figure 2a. Peak insulin concentration was significantly higher for glulisine than for aspart (geometric mean of 534 vs. 363 pmol/l; p < 0.0001; figure 2b). Although *T*_max_ tended to be longer with glulisine (median of 120.0 vs. 93.0 min), this difference was not significant (p = 0.5133). Glulisine was associated with significantly higher AUCs for all four measurement durations (0–1, 0–2, 0–4 and 0–6 h; all: p < 0.0001), with point estimates for mean ratios (glulisine/aspart) ranging from 155% (90% CI: 141–171) for AUC_0–6 h_ to 197% (90% CI: 157–248) for AUC_0–1 h_. In terms of AUC ratios, only AUC_0–1 h_/AUC_0–6 h_ was significantly different between the groups, with the value of this ratio for glulisine being 127% of the equivalent ratio for aspart (90% CI: 106–152; p = 0.0340; Table 2).

### Hypoglycaemia and Safety Parameters

A total of 13 (36.1%) subjects given glulisine and 16 (43.2%) subjects receiving aspart experienced an episode of hypoglycaemia (blood glucose <70 mg/dl with or without symptoms). Among these, 10 and 15 subjects, respectively, experienced an episode of hypoglycaemia 3–6 h after the insulin administration. The remaining episodes occurred 30, 110 and 135 min after glulisine administration and 60 min after aspart administration. Five and eight subjects, respectively, experienced an episode of hypoglycaemia with blood glucose levels below 56 mg/dl. None of the episodes was considered to be severe nor required intervention.

Five treatment-emergent adverse events were reported in four subjects, including injection-site pain (glulisine, one; aspart, one), headache (glulisine, one; aspart, one) and nausea (aspart, one). None of the adverse events was reported as serious.

## Discussion

This two-way crossover study is the first to compare the PK/PD profiles of glulisine and aspart in people with T2DM, given a standard meal under identical baseline plasma glucose concentrations. During the first hour following insulin injection, the absolute plasma glucose concentration was significantly lower after administration of glulisine than with aspart (p = 0.0455). Furthermore, the peak glucose concentration was also significantly lower after glulisine administration than after aspart (p = 0.0337). When considering the overall duration of the study, however, the plasma glucose levels and glucose excursions were similar between the two rapid-acting insulin analogues.

Care must be taken when interpreting the PK data, owing to the different assays used for each insulin analogue. As analogue-specific assays were used for determination of aspart and glulisine, the PK data were normalized to a percentage of *C*_max_ so that the data for the two analogues could be compared. Although there was no difference between groups over the study duration, there was a statistically significant difference in the measured mean insulin concentration over the first 20 min (figure 2b). The C-peptide and NEFA levels throughout the 6-h period were comparable in both groups, indicating that the results were not influenced by changes in endogenous insulin secretion and that both insulins have similar effects on carbohydrate utilization.

Overall, these findings are consistent with previous results obtained in a similar study comparing glulisine and lispro in obese subjects with T2DM [9], which also showed a lower maximum PPG excursion with glulisine. The findings are also consistent with the PD data observed in a study in healthy individuals [13]. These PK and PD differences could be related to the zinc-free formulation of glulisine, which, along with the structural modifications, help to prevent dimerization. Indeed, these changes facilitate the rapid uptake of glulisine from the subcutaneous depot after injection [5,6]. The addition of zinc to the rapid-acting analogues lispro and aspart formulations is necessary to prevent the formation of fibrils [5,16] and to promote the formation of stable hexameric and higher-order aggregates [17,18].

Excess adiposity can adversely affect the PK and PD properties of RHI [19–21]. Indeed, the site of injection may influence the PK and PD of short-acting insulins because body regions with greater skin thickness may show protracted absorption [22]. For example, ter Braak et al. reported that the *C*_max_ and *T*_max_ values for insulin (lispro and human insulin) varied between the two types of insulin and between the three injection sites (abdominal, deltoid and femoral sites) [22]. However, in that study, lispro was consistently associated with better PK and PD parameters vs. RHI, irrespective of the site of injection. Based on the results of the present study in obese individuals with T2DM and other studies in lean to obese subjects without diabetes, it transpires that the onset of action of the rapid-acting insulin analogues is not delayed in obese subjects when using a specific injection site [8]. Unfortunately, in both studies, the actual subcutaneous fat thickness was not assessed and BMI *per se* may not be a good marker for subcutaneous fat at the injection site.

Overall, the findings of the present study must be considered in light of the exploratory nature of this study and small sample size. It must also be noted that a strictly defined meal size and content and a fixed insulin dose were used in this study. Therefore, the results should not be generalized to the population as a whole because meal size and content and insulin doses will vary not only between individuals but also according to meals. However, dose proportionality of glulisine has been described in individuals with T1DM [11] and it is possible that a similar effect may be seen in individuals with T2DM; thus, prospectively altering the insulin dose based on meal content may be more appropriate than a predefined titration algorithm for some individuals [23]. In terms of PD, a similar pattern is likely to be seen to that observed in this study, but will clearly depend on the relative carbohydrate and fat content, aside from the effects of insulin resistance in individuals with T2DM.

In conclusion, this study, involving obese subjects with T2DM, showed that, at identical doses, glulisine was associated with a lower plasma glucose level than aspart during the first postprandial hour, in combination with significantly higher glulisine concentrations and when administered by bolus subcutaneous injection. During the remaining period of the test, there were no differences in the glucose profiles and glulisine levels were higher than aspart. Taken together, the lower early and late AUCs for glulisine support the earlier impact of glulisine, compared with aspart, on the PPG profile in response to a standard test meal.
